# Specific microbiome patterns and their association with breast cancer: the intestinal microbiota as a potential biomarker and therapeutic strategy

**DOI:** 10.1007/s12094-024-03554-w

**Published:** 2024-06-18

**Authors:** Alba Amaro-da-Cruz, Teresa Rubio-Tomás, Ana I. Álvarez-Mercado

**Affiliations:** 1 Department of Chemical Engineering, Faculty of Science, University of Granada, 18071 Granada, Spain; 2 Institute of Molecular Biology and Biotechnology, Foundation for Research and Technology-Hellas, Heraklion, Crete Greece; 3 Instituto de Investigación Biosanitaria ibs.GRANADA, Complejo Hospitalario Universitario de Granada, 18014 Granada, Spain; 4 Institute of Nutrition and Food Technology, Biomedical Research Center, University of Granada, 18016 Armilla, Spain; 5 Department of Pharmacology School of Pharmacy, University of Granada, 18071 Granada, Spain

**Keywords:** Breast cancer, Microbiome, Biomarkers, Therapy, Microbiota, Chemotherapy

## Abstract

Breast cancer (BC) is one of the most diagnosed cancers in women. Based on histological characteristics, they are classified as non-invasive, or in situ (tumors located within the milk ducts or milk lobules) and invasive. BC may develop from in situ carcinomas over time. Determining prognosis and predicting response to treatment are essential tools to manage this disease and reduce its incidence and mortality, as well as to promote personalized therapy for patients. However, over half of the cases are not associated with known risk factors. In addition, some patients develop resistance to treatment and relapse. Therefore, it is necessary to identify new biomarkers and treatment strategies that improve existing therapies. In this regard, the role of the microbiome is being researched as it could play a role in carcinogenesis and the efficacy of BC therapies. This review aims to describe specific microbiome patterns associated with BC. For this, a literature search was carried out in PubMed database using the MeSH terms “Breast Neoplasms” and “Gastrointestinal Microbiome”, including 29 publications. Most of the studies have focused on characterizing the gut or breast tissue microbiome of the patients. Likewise, studies in animal models and in vitro that investigated the impact of gut microbiota (GM) on BC treatments and the effects of the microbiome on tumor cells were included. Based on the results of the included articles, BC could be associated with an imbalance in the GM. This imbalance varied depending on molecular type, stage and grade of cancer, menopause, menarche, body mass index, and physical activity. However, a specific microbial profile could not be identified as a biomarker. On the other hand, some studies suggest that the GM may influence the efficacy of BC therapies. In addition, some microorganisms and bacterial metabolites could improve the effects of therapies or influence tumor development.

## Background

Breast cancer (BC) is one of the most frequently diagnosed tumors in women worldwide. Its incidence has increased in recent decades. Although advances in diagnosis and treatment have improved survival rate, it remains one of the leading causes of cancer death in women [1].

BC is a multifactorial disease whose development involves genetic and environmental components that contribute to the complexity of its treatment and management. A wide variety of risk factors associated with BC have been identified: genetic, such as BRCA1/2 mutations, and environmental, such as alcohol intake, smoking, breast tissue density, sedentary lifestyle and obesity. However, the main risk factor is hormonal exposure throughout life, including physiological changes associated with puberty, menarche, pregnancy, menopause, hormonal contraceptives and hormone replacement therapy. In this context, the risk of BC is directly related to elevated levels of endogenous estrogens and differences in estrogen metabolism, especially in postmenopausal women [2, 3].

Several classifications have been developed to group BC. Based on histological characteristics, there are non-invasive (or in situ) and invasive BC. While non-invasive BC is referred to tumors contained in the milk ducts or lobules (such as ductal or lobular carcinoma in situ), invasive BC means that cancer has spread into the surrounding tissues or other body areas. In this case, BC can be ductal carcinoma no special type, or lobular carcinoma. Over time, in situ carcinomas may become invasive BC [4]. Invasive BC can be divided into different subtypes based on the expression of biomarkers, such as estrogen receptor (ER), progesterone receptor (PR), human epidermal growth factor receptor 2 (HER2) and Ki67 antigen [2, 5, 6]. Perou and Sorlier [7] classify BC into four subtypes: luminal A and luminal B (expressing ER), HER2-enriched and basal-like. However, clinical practice uses a surrogate classification based on histological and molecular characteristics (Table 1). The progression of BC is divided into four stages based on the TNM system (classification of malignant tumors), which considers the size of the primary tumor (T), lymph node involvement (N) and metastasis (M). Similarly, tumor differentiation, percentage of tubular formation, nuclear pleomorphism and mitotic activity establish the grade of the disease. Staging system, grade assessment and analysis of biomarkers have prognostic and predictive value in BC [2, 5]. Table 1 summarizes BC classifications and their main characteristics [4, 9].

**Table 1:** BC classification and main characteristics

Intrinsic subtypes	Surrogate intrinsic subtypes	Biomarkers	Frequency	Therapy	Prognosis
Luminal A	Luminal A-like	ER (+) PR (+) HER2 (−) Ki67 (↓)	30–70%	Endocrine therapy Chemotherapy	Good
Luminal B	Luminal B-like HER2-	ER (+) PR (+) HER2 (−) Ki67 (↑)	15–20%	Endocrine therapy Chemotherapy Targeted therapy	Intermediate
	Luminal B-like HER2+	ER (+) PR (−/+) HER2 (+) Ki67 (↓/↑)		Endocrine therapy Chemotherapy Targeted therapy	Intermediate
HER2-enriched	HER2-enriched (non-luminal)	ER (−) PR (−) HER2 (+) Ki67 (↑)	10–15%	Targeted therapy Chemotherapy	Intermediate
Basal-like	TNBC	ER (−) PR (−) HER2 (−) Ki67 (↑)	10–20%	Chemotherapy PARP inhibitors	Poor

*ER* estrogen receptor, *PR* progesterone receptor, *HER2* human epidermal growth factor receptor 2, *TNBC* triple-negative breast cancer, *PARP* poly(ADP ribose) polymerase ↑ means high expression and ↓ means low expression

The diagnosis of BC is based on the triad of clinical assessment, imaging test and biopsy. The classic imaging test is mammography although it has low sensitivity (25–59%) in young women with dense breasts. In all cases, the diagnosis is confirmed by biopsy [4, 8].

Treatment of BC is based on four main strategies: surgery, radiotherapy, systemic therapy and immunotherapy. Radiotherapy can be used as adjuvant or palliative therapy. Systemic therapy is administered as adjuvant or neoadjuvant and includes chemotherapy, endocrine hormone therapy and biological or targeted therapy [2]. The choice of systemic therapy largely depends on the molecular subtype, stage and grade of BC (Table 1).

Early detection, determination of prognosis and prediction of response to treatment are essential tools for managing BC and reducing its incidence and mortality in the population, as well as promoting personalized therapy for patients [9]. Moreover, more than half of BC cases are not related to any known risk factor [10]. Thereby, research into new biomarkers for diagnostic, prognostic and predictive value has emerged in recent years. In addition, the severity of side effects caused by the existing treatments, and the development of resistance and relapse, highlights the need to develop new therapeutic agents or strategies that improve treatment efficacy.

## Human microbiome

The human body harbors trillions of commensals, symbiotic and pathogenic microorganisms (including bacteria, archaea, viruses, fungi, and protozoa) that comprise the human microbiota. Although this term is commonly used as a synonym for microbiome, the latter encompasses the taxonomy and abundance of microorganisms present in a particular environment (microbiota), their genetic material and their metabolites [11]. The microbiome is a dynamic ecosystem that develops upon birth under the influence of maternal microbiota and environment and varies throughout life, both between and within individuals. The microbiota colonizes different habitats within the human body (gut, oral cavity, vagina, skin, etc.) and its structure widely differs depending on the niche it occupies [3]. Breast tissue hosts a community of bacteria that contributes to maintaining healthy breast tissue by stimulating resident immune cells. In the female mammary gland, milk has been shown to contain bacterial species, ostensibly reaching the ducts from the skin. The phylum with the highest abundance in breast tissue was *Proteobacteria* [12].

The perfect balance in this complex community is known as eubiosis. Alteration or imbalance in the composition of the microbiota (dysbiosis) can trigger harmful effects on human health, leading to a variety of pathological conditions, such as inflammatory bowel disease, diabetes, autoimmune diseases and even some types of cancer [13].

Gut microbiota (GM) is the most widely studied and best characterized in the human body. The main phyla comprising it are *Firmicutes, Bacteroidetes, Actinobacteria, Proteobacteria* and *Verrucomicrobia*. Its distribution varies throughout the gastrointestinal tract depending on the environment [14]. GM composition is defined by various internal and external factors, such as age, race, diet, stress, maternal colonization, host genetics and exposure to antibiotics and xenobiotics [3].

In eubiosis, GM contributes to maintaining the body’s homeostasis and exerts a wide set of beneficial effects on human health. First, GM maintains the intestinal barrier function by strengthening the tight junctions between intestinal epithelial cells and stimulating mucus production. In addition, it stimulates the secretion of immunoglobulin A (sIgA) by the immune cells in the intestine. Second, it competes with pathogenic microorganisms for attachment to the intestinal mucosa (competitive exclusion) or directly prevents attachment of pathogens to the intestinal mucosa. Third, GM produces a wide variety of molecules with diverse biological activities, including short-chain fatty acids (SCFAs), such as acetate, propionate and butyrate, which act as an energy source for intestinal epithelial cells; vitamins, such as K, cobalamin, biotin and folic acid; hormones, such as catecholamines; and neurotransmitters, such as acetylcholine, serotonin, and dopamine. Additionally, some commensal microorganisms produce peptides with antimicrobial activity (bacteriocins such as lactin) and compounds with antifungal activity (such as benzoic acid). Finally, it modulates the immune system by interacting with antigen-presenting cells (such as dendritic cells) or by interacting with the toll-like receptor (TLR) signaling cascade, among other mechanisms. This pathway allows commensal microbiota to trigger a T cell-mediated immune response against cancer cells [15].

## Microbiota and breast cancer

### The role of microbiota in carcinogenesis

Only a percentage of women with genetic predisposition or exposure to known environmental risk factors develop BC, and more than half of cases are unrelated to known risk factors [10]. In this context, the growing evidence for the dual role of the human microbiome in human health and disease has prompted the investigation of the gut and breast microbiome in BC, hypothesizing that dysbiosis could be an additional risk factor.

Alterations in the structure of GM and the functions exerted by the microorganisms can affect the development and progression of BC through various mechanisms. Some bacteria are capable of directly inducing carcinogenesis. In this regard, *Helicobacter pylori* is considered the only direct carcinogenic bacterium in humans and is responsible for gastric adenocarcinomas. However, the potential of other bacteria (“oncomomicrobes”) to induce cancer via genotoxic-mediated mutagenesis through toxins and virulence factors has also been unraveled [14]. Regarding BC, Parida et al*.* [16] reported that intestinal colonization by enterotoxigenic *Bacteroides fragilis*, which secretes the *B. fragilis* toxin, affects epithelial hyperplasia in the mammary gland. Furthermore, in vitro treatment of MCF-7 cells with this toxin before cell injection into mice significantly increases the rate of tumor growth and metastasis.

**Fig. 1:**
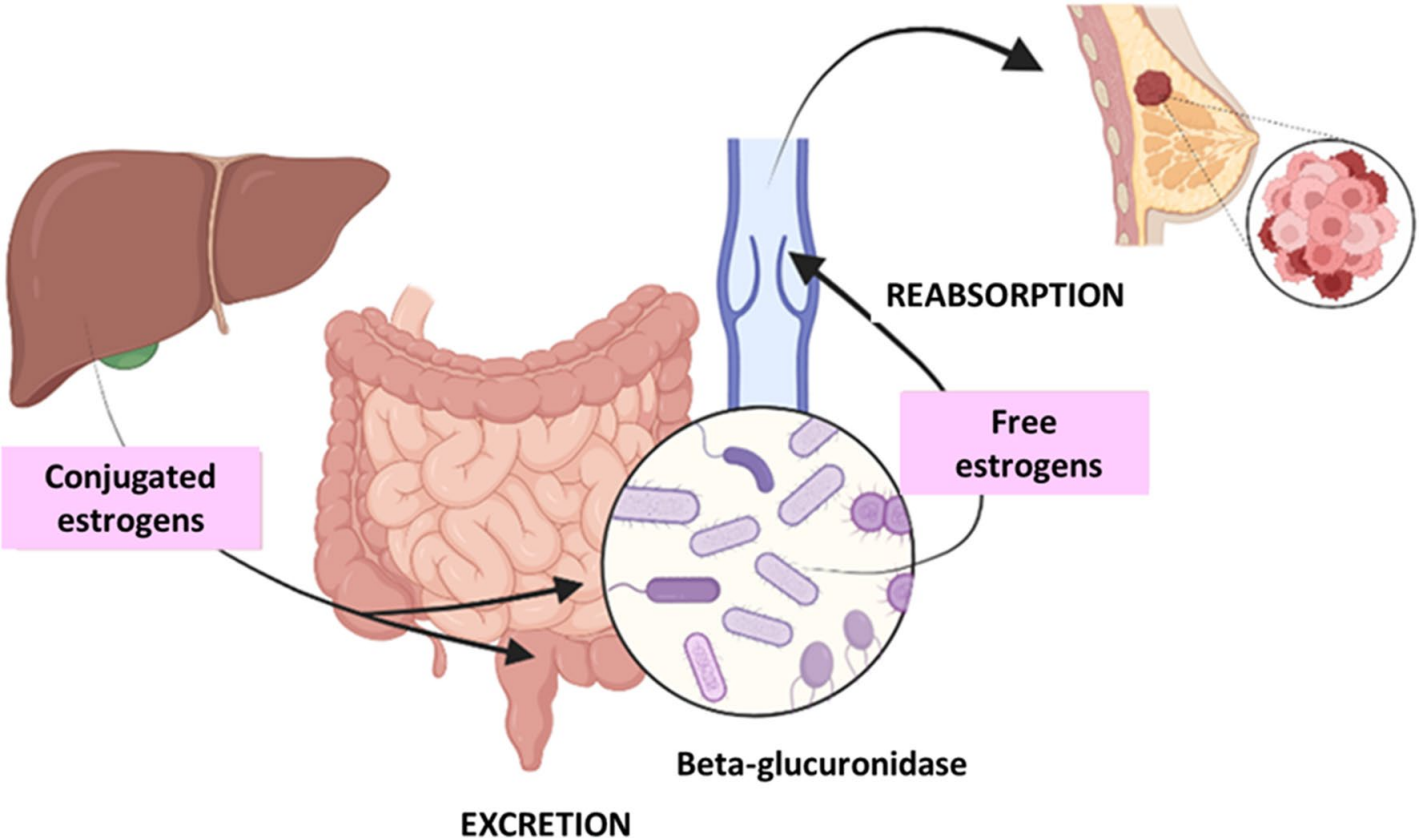
Enterohepatic metabolism of estrogens and the impact of the estrobolome

As mentioned above, hormone exposure is one of the major factors associated with BC development, especially in postmenopausal women. In this context, the estrobolome is particularly important, which refers to the set of microbial genes whose products are involved in estrogen metabolism, such as the enzymes glucuronidases, glucosidases and dehydrogenases [16]. Therefore, alterations in the microbiota/estrobolome can lead to elevated levels of circulating estrogens and their metabolites, increasing the risk of BC. Estrogen metabolism occurs in the liver, where are conjugated and excreted into the gastrointestinal lumen through the bile. There, a fraction is deconjugated by the action of bacterial β-glucuronidase and finally reabsorbed as free estrogens through the enterohepatic circulation, being distributed to organs such as the breast (Fig. 1). Additionally, estrogen-like metabolites that could have carcinogenic potential can be produced in the intestine. Moreover, bacterial β-glucuronidases could be involved in the deconjugation of xenobiotics and/or xenoestrogens, leading to their reuptake through the enterohepatic pathway, thereby increasing their half-life and availability in the organism. Bacteria with β-glucuronidase enzymes are found in two dominant subgroups, the *Clostridium leptum* cluster and the *C. coccoides* cluster, which belong to phylum *Firmicutes*. Moreover, the *Escherichia/Shigella* bacterial group, member of the phylum *Proteobacteria*, also possesses β-glucuronidases [3, 10, 15].

**Fig. 2:**
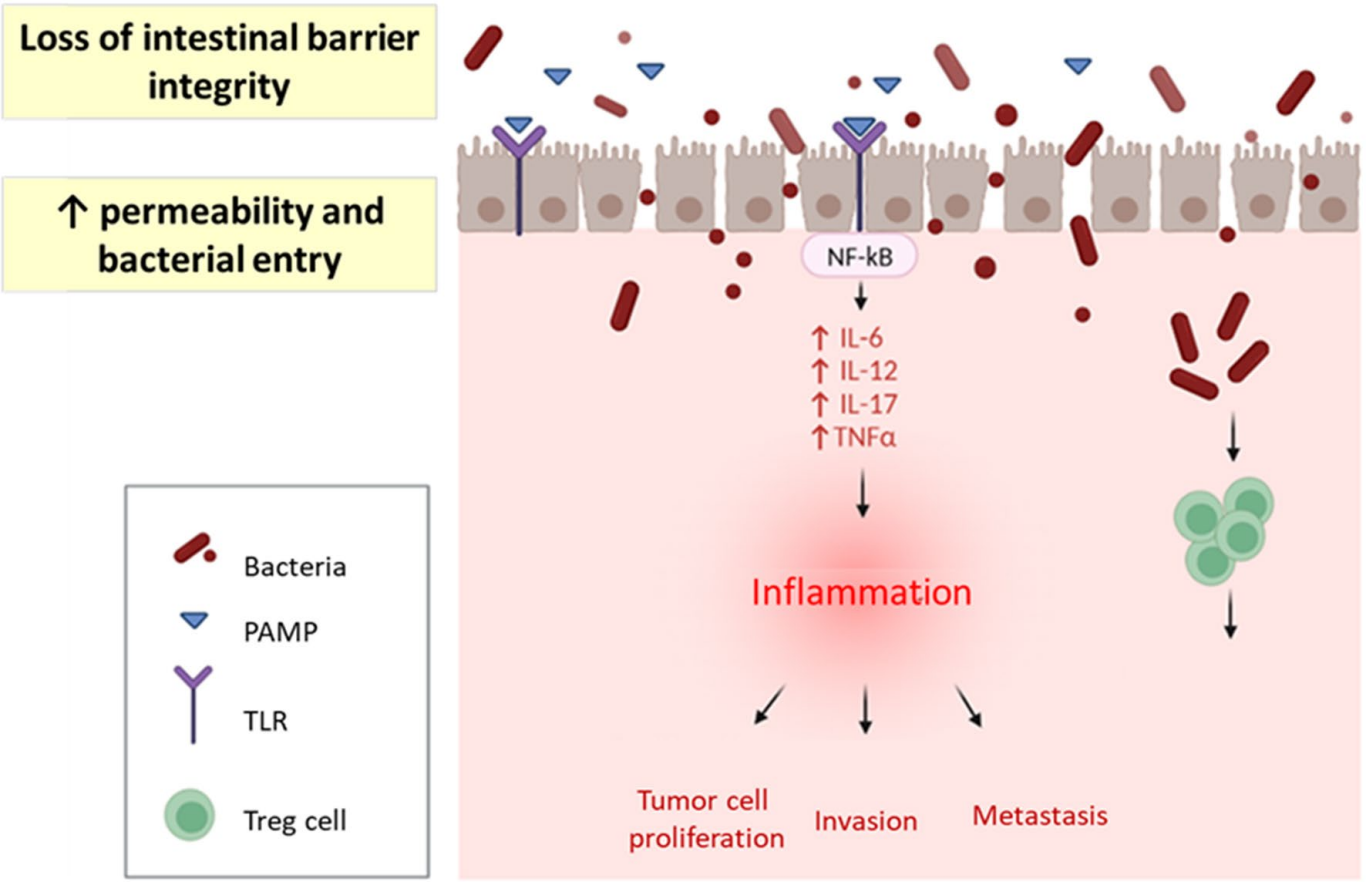
Potential mechanisms by which microbiota modulates tumor microenvironment and favors the development of BC

The immune system plays a key role preventing carcinogenesis and activation of the immune system has been observed upon treatment, especially in HER2+ and triple-negative BC [17, 18]. On the one hand, CD8+ T cells can directly kill cancer cells and their presence is associated with better prognosis. However, FOXP3+ CD4+ regulatory T cells mediate immunotolerance and correlate with poor prognosis. In BC, the proportion of regulatory T cells (Treg) increases with the disease stage and is associated with relapse and decreased survival. Due to the immunomodulatory capacity of the microbiota, changes in the abundance of specific bacteria could likely lead to increased production of Treg cells (Fig. 2). Furthermore, in animal models, some bacterial metabolites, such as butyrate and propionate, reduce inflammation via altering colonic regulatory T cells [10, 19]. Therefore, the microbiota and its metabolites can modulate the local immune microenvironment.

Microbiota can contribute to the development of BC by inducing a state of chronic inflammation. When the integrity of the intestinal barrier is lost, gut bacteria can upregulate TLRs and activate the nuclear factor-κB (NF-κB) pathway, which regulates inflammation and has been implicated in cancer. Pathogen-associated molecular patterns (PAMPs) are recognized by innate immune cells through pattern recognition receptors (PRRs), such as TLRs and NOD-like receptors (NLRs). These PAMPs are essential components of pathogens, such as lipopolysaccharide, flagellin, lipoteichoic acid and peptidoglycans. When TLRs recognize PAMPs, they activate the production of proinflammatory cytokines. Chronic TLR activation promotes tumor cell proliferation and enhances the mechanisms of invasion and metastasis through the regulation of cytokines, metalloproteinases and pro-inflammatory integrins [10, 20]. Consequently, chronic inflammation affects both the initiation and the progression of BC due to the constant presence of inflammatory cytokines and the recruitment of immune system cells, such as Tregs, which, as mentioned above, decrease the immune response promoting immune evasion by the tumor (Fig. 2).

Furthermore, changes in epigenetic marks and dietary patterns also affect microbiota composition and influence carcinogenesis. Modifications of epigenetic marks, such as those inducing the inactivation of tumor suppressor genes, are observed in patients with BC. In this line, the gut microbiome can contribute to this dysregulation through several mechanisms. Specific bacterial metabolites, such as SCFAs (butyrate and propionate), folates and biotins, can epigenetically modify gene expression. Similarly, microbiota synthesizes enzymes that induce epigenetic changes and contribute to the absorption of minerals that act as cofactors for these enzymes. In addition, the content and quality of the diet is particularly related to BC and is an important modulator of the diversity and structure of the microbiota [10]. Data from two large cohort studies showed that consuming more polyunsaturated and vegetable fats was linked to a decreased risk of hepatocarcinoma. This was related to the replacement of animal or dairy fats with vegetable fats or replacing saturated fats with monounsaturated or polyunsaturated fats [21].

### The role of microbiota in breast cancer therapeutic approaches

Treatments against BC, such as radiotherapy, systemic therapy or immunotherapy, can alter the microbiota of patients, which, in turn, may affect the efficacy and side effects of treatments. On the other hand, GM can modulate cancer progression through the synthesis of antitumor compounds and the regulation of immune response and inflammatory pathways of the host [15]. Thus, the combination of these mechanisms may explain the impact of the microbiota on the efficacy of different cancer therapies.

Indeed, it is known that GM can participate in the metabolism of a wide range of drugs used in chemotherapy, thus modulating the response to treatment. For example, gemcitabine is a pyrimidine antagonist whose antitumor activity is based on its intracellular activation and subsequent degradation to an inactive compound by the enzyme cytidine deaminase. Studies in mice has shown that resistance to gemcitabine may be due to increased metabolic degradation of the drug due to the expression of an isoform of the bacterial enzyme cytidine deaminase, observed mainly in the *Gammaproteobacteria* class. Hence, the combination of gemcitabine with ciprofloxacin favors the antitumor activity of the drug; this synergism is caused by the bacterial inhibition exerted by the antibiotic. Furthermore, 5-fluorouracil is a thymidylate synthase inhibitor whose therapeutic use is limited due to the development of resistance and its gastrointestinal side effects. Preclinical trials in mice have shown that administration of 5-fluorouracil in combination with a cocktail of antibiotics decreases antitumor efficacy, while supplementation with probiotics significantly enhances anti-cancer effects [15].

Endocrine hormone therapy is used against hormone receptor-positive BC and it target the ER directly or the estrogen synthesis. The main types of endocrine therapy are selective ER modulators, selective modulators ER degraders, and aromatase inhibitors (AI) [22]. Nevertheless, there are few works addressing the role of GM in hormone therapies. In this regard, Lasagna et al. [23] carried out a monocenter observational case–control study in which they discovered that postmenopausal BC women who respond to AI had different fecal microbiota abundance than those resistant to AI therapy. In particular, *Veillonella* genus were enriched in the GM of patients resistant to AI. Although its implication in BC remains undetermined, the presence of *Veillonella* species in the GM of patients treated with CAR T cells have been associated with poor prognosis [24].

Immunotherapy is based on the use of immune checkpoint inhibitors (ICIs), molecules that block specific immune regulatory pathways to enhance the antitumor immune response. ICIs are monoclonal antibodies that target receptor molecules on the surface of T cells, such as cytotoxic T cell antigen 4 (CTLA-4) and the programmed cell death 1 (PD-1) receptor or PD-1 ligands (PD-L1 and PD-L2). By dysregulating the immune system, these molecules cause a wide range of side effects. Moreover, some patients do not benefit from treatment (primary resistance) or show no improvement in disease progression (secondary resistance). Furthermore, there is evidence that in some cases ICIs can favor tumor development. Consequently, studies have been carried out to identify predictive factors of the efficacy of these treatments, as well as strategies to overcome treatment resistance. Some of them have shown that the GM composition modulates the activity, efficacy and toxicity of ICIs. For example, anti-PD-L1 antibody shows more efficacy for the treatment of melanoma in mice when GM is enriched in *Bifidobacterium* species. Oral administration to patients of a bacterial cocktail of these species together with the anti-PD-L1 antibody specifically increases the T cell response and blocks the growth of melanoma, whereas if the treatment is combined with antibiotics, the survival rate is reduced [15].

### Probiotics, prebiotics and breast cancer

Probiotics are defined as “strictly selected live strains of microorganisms that, when administered in adequate amounts, confer beneficial effects on the health of the host” [25]. Many studies have been carried out in mouse models to investigate the effects of probiotics on BC. Most studies are based on the administration of strains of the genus *Lactobacillus,* such as *L. helveticus* R389, *L. acidophilus* and *L. reuteria*, and their ability to prevent and control cancer progression is related to the modulation of the host immune system. However, clinical studies of probiotics in BC patients are very limited [10].

A prebiotic is defined as “a substrate that is selectively utilized by host microorganisms conferring a health benefit” [25]. They are mainly indigestible dietary fiber compounds that, when combined with harmful and carcinogenic substances in the intestine, promote their breakdown and the growth of probiotics, inhibiting the proliferation of pathogenic bacteria and the production of carcinogens. The effect of different prebiotics has been investigated in the context of BC, such as plant-based lignans, SCFAs or polyphenols. GM transforms plant-based lignans (present in soybeans, flax and sesame seeds, etc.) into phytoestrogens, estrogen-like compounds. Phytoestrogens act against BC at concentrations above 10 µM by inhibiting the synthesis and metabolism of estrogens and inducing antiangiogenic, antimetastatic and epigenetic effects. SCFAs produced by bacterial fermentation of dietary fiber also have anticancer effects, especially butyrate. Butyrate can reduce the viability of MCF-7 tumor cells. Finally, dietary polyphenols are bio transformed by GM into derivatives with increased bioavailability. In addition, dietary polyphenols can modulate the composition of the gut microbial community, inhibiting the proliferation of pathogenic bacteria and stimulating the activity and proliferation of beneficial bacterial species [10].

## Rationale and hypotheses

There is an increasing number of studies characterizing the GM of BC patients and healthy women to describe specific microbial signatures of BC. Broadly, most studies define the microbiota based on three characteristics: (1) alpha (α) diversity, which refers to the diversity within a community of microorganisms and includes parameters, such as richness and uniformity; (2) beta (β) diversity, which refers to the differences between communities; and (3) taxonomic composition (measured in operational taxonomic units, OTUs), which reports on the abundance (absolute or relative) of specific members of the community [13]. Recently, recent studies have also analyzed breast microbiota composition.

## Objective

Considering all of the above information, the general aim of this review is to describe specific microbiome patterns that could be associated with BC. The specific aims of the review are (1) to describe the differences between the GM of women with BC and healthy controls, (2) to define possible microbial profiles that could be used as non-invasive biomarkers of BC, (3) to provide insight into the influence of the GM profile in the treatment of BC and (4) to investigate the potential application of microorganisms as probiotics.

## Materials and methods

### Search strategy

A literature search has been carried out using the PubMed database. To use appropriate English vocabulary and terminology, the following MeSH terms from the MeSH medical database were used: “Breast Neoplasms” and “Gastrointestinal Microbiome”. To optimize and refine the search, the above terms were combined with the Boolean operator “AND” to return all results containing the specified terms.

### Inclusion criteria

The following eligibility criteria were applied to the literature search: (1) articles published between 2016 and 2022 to accomplish an updated literature review, (2) documents with full text available, and (3) articles published in English.

### Study selection and information collection

**Fig. 3:**
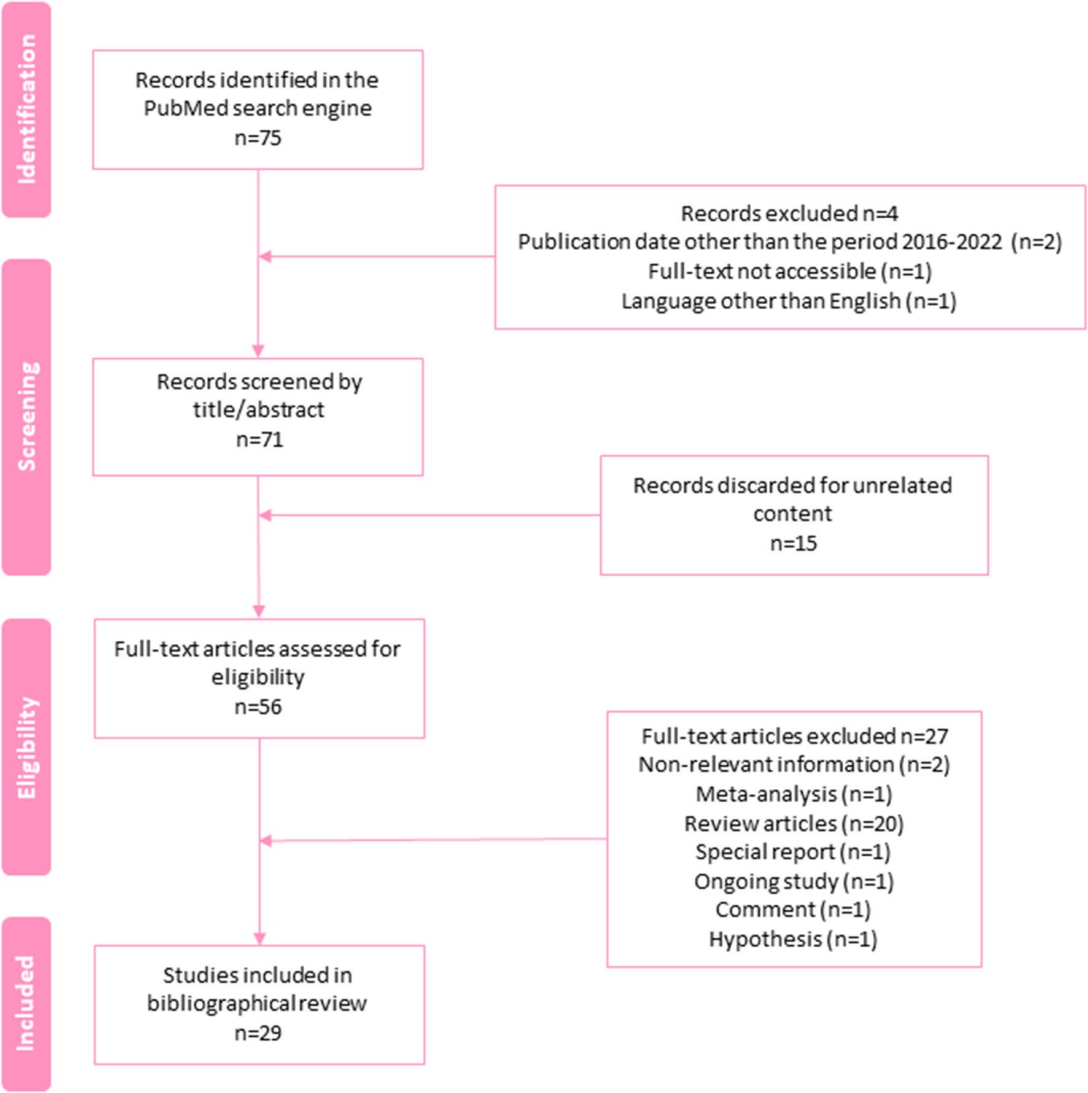
Flowchart of the literature selection process

The literature search using the mentioned MeSH terms combined with the Boolean operator “AND” provided an initial result of 75 publications (Fig. 3). Application of the aforementioned inclusion criteria reduced the search to 71 articles. The selection of publications that met the inclusion criteria was carried out by two independent researchers by reviewing the title and abstract, to discard those articles that did not contain relevant information to achieve the proposed objectives. Thus, 15 articles were discarded. To ensure that only publications that provided information of interest were included, an exhaustive reading of full-text selected articles was carried out. This assessment allowed us to finally include 29 publications.

## Results and discussion

A large proportion of the articles selected have in common the aim of characterizing the gut microbiome or breast tissue of BC patients. The selected studies analyze patients with different molecular types, stages, and grade of cancer, as well as specific groups of patients: premenopausal, postmenopausal, overweight, or obese, undergoing chemotherapy, with benign breast lesions, with non-malignant breast disease or with benign breast disease [26–42]. We also include a group of studies in animal models [26, 36, 43–52] and cell cultures [28, 33, 40, 53, 54] that focused on the impact of the GM on various treatments against BC, as well as on determining the effect of the GM or its metabolites on tumor cells.

Nowadays, the two most extensively used metagenome sequencing strategies are shotgun and the 16S rRNA. Both are being used to catalog the human microbiome in health and disease and to study microbial communities of medical, pharmaceutical, or biotechnological relevance [55]. However, it is noteworthy that we have not found any works using full-length sequencing. This technique is performed to determine the complete sequence of the protein-coding as well as the non‐coding parts of the mRNA which allows us to reach species and regions to genus [56].

For instance, a comparative analysis by Durazzi et al. [57] showed that the use of 16S rRNA technique sequenced partly the GM community detected by shotgun and that genera detected by shotgun sequencing are biologically meaningful even when less abundant.

All the included studies, their main characteristics and findings are summarized in Tables 2, 3 and 4.

**Table 2:** Human studies on microbiota and breast cancer

Publication	Aim	Type of study	Sample	Type of sample	Methodology	Main findings
Luu et al. (2017) [29]	Investigation of the relation between fecal microbiota composition and clinical characteristics in patients with early-stage BC	N/A	31 patients	Stool	qRT-PCR of ARNr 16S gene	In overweight/obese patients ↓ *Firmicutes*, *Faecalibacterium prausnitzii, Blautia* sp. and *Eggerthella lenta* compared to patients with normal BMI In stage II/III patients ↑ *Bacteroidetes*, *Clostridium coccoides* cluster, *C. leptum* cluster, F*. prausnitzii* and *Blautia* sp. compared to stage 0/I ↑ *Blautia* sp*.*, the higher the tumor grade
Goedert et al. (2018) [31]	Determination of the association of postmenopausal BC with estrogen levels, the inflammation marker PGE-M, and fecal microbiota coated or not with mucosal IgA	Case–control	48 postmenopausal patients (88% RE+) and 48 controls	Stool and urine	Sequencing of the V4 region of the 16S rRNA gene (Illumina MiSeq), HPLC/MS and radioimmunoassay	PGE-M levels were not associated with tumor status, estrogen levels, or α-diversity In patients IgA-positive GM had ↓ α-diversity and ↑ metabolic pathways of immune system diseases; while IgA-negative GM had ↓ α-diversity unrelated to estrogen levels
Zhu et al. (2018) [32]	Comparison of GM and its functional capacities between BC patients and controls	Case–control	18 premenopausal patients and 25 controls; 44 postmenopausal patients and 46 controls	Stool	Shotgun metagenomic sequencing (Illumina Hiseq × 10)	Premenopausal patients: ↑ α-diversity, tendency to ↓ β-diversity and differences at the gut metagenome level. The relative abundance of species did not differ significantly between the groups Postmenopausal patients: ↑ α and β-diversity, differences in the gut metagenome and abundance of 45 species
Ma et al. (2020) [40]	Evaluation of changes in GM composition and metabolomics in BC and to investigate the effect of *Faecalibacterium prausnitzii* on BC cells	N/A	25 BC patients and 25 patients with benign breast disease	Stool and blood	16S rRNA gene sequencing (Illumina HiSeq) and metabolomics (LC–MS)	Patients with BC: ↓ α diversity and different β diversity, ↓ a relative abundance of *Firmicutes* (*Faecalibacterium)* and *Bacteroidetes* and ↑ Verrucomicrobia, *Proteobacteria* and *Actinobacteria* Differences in metabolomic composition between groups *Faecalibacterium* ↓ was negatively related to several phosphorylcholines
Wu et al. (2020) [30]	Investigation of the relation between the gut microbiome, BC and its risk factors	Pilot cross-sectional observational	37 patients	Stool	Sequencing of the V3-V4 regions of the 16S rRNA gene (Illumina MiSeq)	↓ α-diversity in HER2+ patients, with high total body fat, obese or overweight, early menarche and sedentary compared with HER2- patients, low total body fat, normal BMI, late menarche and physically active; respectively HER2+ patients compared to HER2- presented ↓ *Firmicutes* and ↑ *Bacteroidetes*, early menarche compared to late menarche was associated with ↓ *Firmicutes* and a higher grade/stage of the tumor was observed ↑ *Clostridium* and *Veillonella* and ↓ *Erysipelotrichaeceae*
He et al. (2021) [33]	Investigation of changes in GM and its metabolites in premenopausal BC patients	Case–control	54 premenopausal patients and 28 controls	Stool	Sequencing of the V3–V4 regions of the 16S rRNA gene (Illumina MiSeq), and targeted metabolomics	In premenopausal BC: ↑ *Firmicutes*/*Bacteroidetes* ratio, ↓ SCFA-producing bacteria and intestinal levels of SCFA (especially butyrate), ↓ *Pediococcus* and ↑ *Desulfovibrio*
Di Modica et al. (2021) [26]	Investigation of the effect of GM on the immune-mediated antitumor efficacy of trastuzumab in preclinical models of HER2+ and HER2+ BC patients	N/A	24 HER2+ patients undergoing neoadjuvant chemotherapy with trastuzumab	Stool, tumor biopsy	Sequencing of the V3–V4 regions of the 16S rRNA gene (Illumina MiSeq) and gene expression profiling	Patients with complete response to treatment: ↑ α-diversity and *Clostridiales*. β-diversity was correlated with immune pathways relevant to immune cell activation and trastuzumab activity
Terrisse et al. (2021) [36]	Determination of the composition of GM, its modulation by adjuvant treatments and its relevance in the efficacy and side effects of therapies against BC	Case–control	76 pre-chemotherapy patients, 45 post-chemotherapy patients and 336 controls	Stool and plasma	Shotgun metagenomic sequencing and metabolomics	β-diversity of GM was related to the tumor stage, grade and involvement of the axillary nodes, as well as post-chemotherapy neurological side effects. The functional pathways associated with these bacterial patterns changed depending on the prognosis
Byrd et al. (2021) [39]	Investigation of the relation of fecal bacteria with BC and non-malignant breast disease	Cross-sectional case–control	379 patients with BC, 102 with non-malignant breast disease and 414 controls	Stool	Sequencing of the V4 region of the 16S rRNA gene (Illumina MiSeq)	An inverse relation between α-diversity of GM and probability of cancer, non-malignant breast disease and molecular grade/subtype of BC Different β-diversity in GM of patients with BC and non-malignant breast disease Association between taxa with roles in estrogen metabolism and immune homeostasis with BC and non-malignant breast disease
Smith et al. (2021) [35]	Characterization of GM in patients with BC or prostate with overweight and obesity	Case–control	14 patients and 14 controls	Stool	Sequencing of the V4 region of the 16S rRNA gene (Illumina MiSeq)	There were no significant differences in α-diversity between groups, but there were in β-diversity. In patients ↑ *Allobaculum* and ↓ *Lysobacter*, while in controls ↑ *Agrobacterium*
Bobin-Dubigeon et al. (2021) [27]	Characterization of the fecal microbiota of patients with early-stage BC	Comparative case–control	25 RE+/RP+ patients and 30 controls	Stool	Sequencing of the V3–V4 regions of the 16S rRNA gene (Illumina MiSeq)	BC patients: ↓ α-diversity and different composition at the phylum and genus level (↑ *Firmicutes*, *Clostridium* cluster IV and cluster XIVa, *Blautia*, *Clostridium* XVIII and *Lachnospira*; ↓ *Bacteroidetes*, *Bifidobacterium* sp., *Odoribacter* sp., *Butyricimonas* sp. and *Coprococcus* sp.)
Saud Hussein et al. (2021) [41]	Determination of the role of the microbiota in the development of BC	Cross-sectional	30 patients and 20 controls with benign breast lesions	Breast tissue and blood	ELISA and bacterial culture	In 20% of the BC tissue samples, microbial growth was observed and the presence of *Escherichia coli* and *Staphylococcus aureus* was determined Difference between women with BC and benign lesions depending on the place of residence ↓ IL-19 in BC than in benign breast lesions
Hou et al. (2021) [34]	Investigation of the gut microbial profile, diagnostic value and functional pathways in premenopausal BC patients	Case–control	100 premenopausal patients and 50 controls; 100 postmenopausal patients and 17 controls	Stool	Sequencing of the V3–V4 regions of the 16S rRNA gene (Illumina MiSeq)	Premenopausal patients: ↓ α-diversity and different β -diversity Identification of 14 BC biomarkers Variation in functional pathways between patients and controls
Esposito et al. (2022) [42]	Characterization of the microbiome of breast tissue in women with BC, comparing tumor and adjacent non-tumor tissue	N/A	34 patients	Tumor breast tissue and healthy adjacent tissue	Sequencing of the V3–V4 regions of the 16S rRNA gene (Illumina MiSeq)	Tumor breast tissue: ↓ α-diversity, ↑ *Proteobacteria* and ↓ *Actinobacteria* (especially *Propionibacterium acnes*) Healthy adjacent tissue: predominance of Propionibacterium and *Pseudomonas*
Zhu et al. (2022) [28]	Investigation of the effect of fecal metabolites on the proliferative activity of malignant mammary tumor	N/A	14 patients and 14 controls	Stool	Non-targeted metabolomic analysis (LC–MS), targeted SCFA analysis, and sequencing of the V3–V4 regions of the 16S rRNA gene (Illumina)	Different metabolites and microbiota between patients and controls. The ↑ abundance of *Rothia* and *Actinomyces* in patients was positively associated with 4-methylcatechol and guaiacol and negatively associated with norvaline
Ma et al. (2022) [38]	Investigation of the composition and function of the GM in patients with BC and benign breast lesions	Cross-sectional	26 patients with BC, 20 patients with benign breast lesions and 20 controls	Stool	Sequencing of the V3–V4 regions of the 16S rRNA gene (Illumina Hiseq 2500)	BC patients: ↓ α-diversity Different β-diversity between groups ↑ *Porphyromonas, Prevotella, Peptoniphilus* and *Megamonas* in BC and ↑ *Escherichia, Lactobacillus* and *Coprobacillus* in benign breast lesions
Bilenduke et al. (2022) [37]	Investigation of the relationship of self-reported cognitive functioning, symptoms of depression and anxiety, and gut microbiome composition in women with BC undergoing chemotherapy	Cross-sectional case–control	21 patients and 14 controls	Stool	Sequencing of the V4 region of the 16S rRNA gene (Illumina MiSeq)	BC patients: different composition (↓ *Akkermansia*) Relationship between greater cognitive deterioration and symptoms of depression with differences in the structure of the gut microbiome of patients

*BC* breast cancer, *N/A* not stated, *RRNA* ribosomal ribonucleic acid, *ER+* estrogen receptor-positive, *RP+* progesterone receptor-positive, *GM* gut microbiota, *HER2+* human epidermal growth factor receptor 2-positive, *HER2−* human epidermal growth factor receptor 2-negative, *BMI* body mass index, *QRT-PCR* quantitative real-time polymerase chain reaction; *RT-PCR* reverse transcriptase polymerase chain reaction, *SCFA* short-chain fatty acids, *IL-19* interleukin 19, *PGE-M* prostaglandin e metabolite, *IGA* immunoglobulin A

↑ means increase and ↓ means decrease

**Table 3:** Studies in animal models on microbiota and breast cancer

Publication	Aim	Cancer type/induction/treatment	Analyzed sample	Main methods	Main findings
Paul et al. (2017) [43]	Investigation of the effect of genistein on the gut microbiome of germ-free mice and its impact on the prevention and treatment of ER- -BC	RAG2^−/−^ athymic germ-free mouse Orthotopic injection of MDA-MB-231 cells FMT of patients before and after chemotherapy and administration of genistein or control diet	Stool, urine, serum, tumor	Sequencing of the V4 region of the 16S rRNA gene (Illumina MiSeq) and metabolomic analysis (LC–MS/MS)	Genistein altered GM composition before tumor induction, contributing to ↑ latency and ↓ tumor volume and weight
Xue et al. (2018) [44]	Determination of the effect of fucoidan on GM and intestinal barrier function in rats with induced BC	Spraque Dawley rat DMBA subcutaneous injection Control group, model and low and high doses of fucoidan (200 and 400 mg/kg by body weight)	Stool, blood, intestinal tissue	Sequencing of the V3–V4 region of the 16S rRNA gene (Illumina MiSeq)	Fucoidan ↑ gut microbiome diversity, *Firmicutes*/*Bacteroidetes* ratio and abundance of SCFA producers such as *Prevotella*; and repaired intestinal barrier function
Shi et al. (2019) [45]	Investigation of the ability of EcN to potentiate TGF-β blockade immunotherapy by modulating GM and the tumor microenvironment in a BC model	Pathogen-free BALB/c mouse Injection of 4T1 cells Control group, EcN (1 × 10^9^ UFC), Gal (45 mg/kg) and Gal + EcN Treatment with an antibiotic cocktail and TMF of mice with tumors and response to EcN	Stool, tumor, blood	Sequencing of the V3–V4 regions of the 16S rRNA gene (Illumina MiSeq)	EcN enhanced the effect of TGF-β inhibitors: ↑ tumor suppression and modulated GM (↑ abundance of *Alistipes shahii*, *Akkermansia muciniphila*, *Bacteroides thetaiotaomicron*, *B. acidifaciens* and *Lactobacillus johnsonii*; ↓ abundance of *Clostridium* spp.)
Rosean et al. (2019) [46]	Determination of the relation of pre-existing dysbiosis of the commensal microbiome with prognosis of RE/RP+ BC	Mouse C57BL/6 Orthotopic injection of 5E5 BRPKp110 or 1E5 PyMT cells or intraductal injection of adenovirus-Cre Induction of dysbiosis through an absorbable, non-absorbable or TMF antibiotic cocktail	Stool, breast tissue and serum	16S rRNA gene sequencing and metabolomic analysis	Commensal dysbiosis ↑ tumor cell dissemination, fibrosis, and collagen deposition within the tissue and tumor microenvironment; generating early inflammation and myeloid infiltration in the breast tissue and tumor
Jian and Fan (2021) [47]	Investigation of the effect of ethanol extracts from *Poria cocos* on the intestinal barrier function and the microbiota in mice with triple-negative BC	Pathogen-free nude BALB/c mouse MDA-MB-231 cell xenograft Control group, model, *Poria cocos* (100 μL *Poria cocos* solution/10 g weight) and positive control (2 mg/kg of cisplatin)	Stool, blood, urine, intestinal tissue	Sequencing of the V3–V4 region of the 16S rRNA gene (Illumina MiSeq)	*Poria cocos* ↑ the integrity of the intestinal barrier, the diversity of GM and the abundance of *Lactobacillus* and *Bifidobacterium*; and ↓ abundance of *Desulfovibrio*, *Mucispirillum*, *S24-7* and *Staphylococcus*
Han et al. (2021) [48]	Determination of the activity against BC of three sweet potato phytosterols in a nude mouse model with ER+ BC	BALB/c nude mouse Orthotopic xenograft of MCF-7 cells Administration of 87.8 mg/kg/day of DLA, DL and DP	Stool, serum, tumor tissue, intestinal tissue, heart, liver, spleen, kidney, lung	Sequencing of the V3–V4 regions of the 16S rRNA gene (Illumina HiSeq)	DLA, DL and DP ↓ tumor growth by ↓ tumor marker levels, altering the expression of cancer-related proteins, and modulating GM and SCFA production
Di Modica et al. (2021) [26]	Investigation of the effect of GM on the immune-mediated antitumor efficacy of trastuzumab in preclinical models of HER2+ BC and HER2+ BC patients	FVB/Ncrl mouse Injection of MI6 HER2+ cells Administration of trastuzumab after vancomycin or streptomycin treatment or TMF of vancomycin-treated mice	Stool, ileum, colon and tumor biopsy	Sequencing of the V3–V4 regions of the 16S rRNA gene (Illumina MiSeq) and gene expression profiling	Antibiotic administration or FMT of vancomycin-treated donors ↓ trastuzumab activity by altering GM composition The TMF of trastuzumab complete responders and non-responders to HER2+ mice recapitulated the trastuzumab response observed in patients
Terrisse et al. (2021) [36]	Determination of GM composition, its modulation by adjuvant treatments and its relevance in the results and side effects of therapies against BC	Mouse C57BL/6 Syngeneic transplantation of AT-3 cells Administration of CTX after humanization by TMF of patients with BC or controls	Stool	Shotgun metagenomic sequencing and metabolomics	The tumor-killing activity of CTX-based chemotherapy was ↓ in TMF mice from BC patients than from controls
Li et al. (2021) [49]	Investigation of the mechanism by which maternal n-3 PUFAs decrease the risk of BC in the offspring in relation to GM	C57BL/6 mouse DMBA administration (65 mg/kg) Standard control diet, a diet supplemented with fish oil (n-3 Sup-FO), with linseed oil (n-3 Sup-FSO), or deficient in n-3 PUFA (n-3 Def) during pregnancy and lactation	Breast tissue, tumor tissue, blood and stool	Sequencing of the V3–V4 region of the 16S rRNA gene (Illumina MiSeq)	Maternal supplementation with n-3 PUFA: ↓ tumor incidence and volume, ↑ IM α-diversity in offspring and ↓ levels of proinflammatory factors IL-1β, IL-6, and TNF-α Diets with n-3 PUFA: ↑ the relative abundances of *Akkermansia, Lactobacillus* and *Mucispirillum*
Shiao et al. (2021) [50]	Investigation of the effect of the bacteriome and mycobiome on the efficacy of radiotherapy against luminal B BC	C57BL/6 mouse Syngeneic injection of E0771 cells Focal irradiation, administration of antibiotic cocktail and focal irradiation, treatment with fluconazole (0.5 mg/mL) or 5-fluorocytosine (1 mg/mL), administration of cefoperazone followed by three doses of C. albicans and gnotobiotic-free mice. fungi after colonization with Schaedler’s altered flora subjected to focal irradiation	Tumor tissue, stool, breast	Sequencing of the 16S rRNA gene and the ITS1 gene (Illumina MiSeq)	The reduction of intestinal fungi by antibiotic administration or gnotobiotic exclusion: ↑ the efficacy of radiotherapy Reduction of bacteria by antibiotics: ↓ response capacity and ↑ commensal fungi
Hossain et al. (2021) [51]	Investigation of the role of obesity in the modulation of the gut microbiome in a triple-negative BC model	Obese FVB mouse Injection of syngeneic cells C0321 Western diet	Stool and mammary tumor	Sequencing of the V4 region of the 16S rRNA gene (Illumina MiSeq) and metagenomic analysis	In obese mice ↑ tumor growth, ↓ α-diversity, *Bacteroides*/*Firmicutes* ratio (highlighting *Alistipes*) and alteration of bacterial metabolic pathways
Loman et al. (2022) [52]	Investigation of the consequences of implantation, growth and resection of orthotopic syngeneic mammary tumors on fecal microbiome composition and intestinal barrier function in relation to systemic inflammation and enteric bacterial translocation in mice	Balb/c mouse Surgical inoculation of the 67NR cell line Simulated surgical control, tumor recipients, and tumor recipients with tumor resection	Stool, colon, spleen and brain tissue	Sequencing of the V4 region of the 16S rRNA gene (Illumina MiSeq)	Breast tumor ↓ the integrity of the colonic barrier by altering the GM (↓ *Lactobacillus* and ↑ *Bacteroides*), which favored the translocation of intestinal bacteria and systemic inflammation

*BC* breast cancer, *rRNA* ribosomal ribonucleic acid, *RH+* hormone receptor-positive, *FMT* fecal microbiota transplantation, *GM* gut microbiota, *CTX* cyclophosphamide, *HER2+* human epidermal growth factor receptor 2-positive, *HER2−* human epidermal growth factor receptor 2-negative, *ER−* estrogen receptor-negative, *DMBA* 7,12-dimethylbenz[a]anthracene, *SCFA* short-chain fatty acids, *DLA* daucosterol linolenate, *DL* daucosterol linoleate, *DP* daucosterol palmitate, *EcN* *Escherichia coli* Nissle 1917, *TGF-β* transforming growth factor β, *Gal* galunisertib, *PUFA* polyunsaturated fatty acids, *IL-1β* interleukin 1β, *IL-6* interleukin 6, *TNF-α* tumor necrosis factor α. ↑ means increase and ↓ means decrease

**Table 4:** In vitro studies on microbiota and breast cancer

Publication	Aim	Cell line/BC model	Methods	Stimulus or treatment	Main findings
Ma et al. (2020) [40]	Evaluation of changes in GM composition and metabolomics in BC and the effect of *F. prausnitzii* supernatant	MCF-7	Western blot, ELISA, cell proliferation, apoptosis and invasion assays	Bacterial supernatant from *F. prausnitzii* grown until stationary phase, lyophilized and diluted in DMEM medium that was used to culture MCF-7 cells for 72 h	*F. prausnitzii* prevents the growth of BC cells through the inhibition of IL-6/STAT3 pathway
Bobin-Dubigeon et al. (2020) [53]	Investigation of the relation between GM and the metabolism of intestinal lipids, and its role in BC	Caco-2 and MCF-7	qPCR, RT-qPCR, capillary gas–liquid chromatography (SGE BP21)	N/A	GM interacts with cellular lipid metabolism and influences the behavior of BC cells
He et al. (2021) [33]	Investigation of changes in GM and its metabolites in patients with premenopausal BC	SKBR3 and MCF-7	Cell culture and viability	0.5 mM, 1.0 mM, and 1.5 mM sodium butyrate or sodium propionate for 48 h	Sodium propionate and sodium butyrate ↓ the activity of SKBR3 and MCF-7 cells in vitro in a dose-dependent manner
An et al. (2021) [54]	Investigation of the effects of extracellular bacterial vesicles from *Klebsiella pneumoniae* in the endocrine therapy of BC	MCF-7	qRT-PCR and Western blot	10 mM tamoxifen, 100 ng/ml K. pneumoniae extracellular vesicles, or 10 mM tamoxifen and 100 ng/ml K. pneumoniae extracellular vesicles for 72 h	*K. pneumoniae*-derived bacterial extracellular vesicles ↑ the antihormonal effects of tamoxifen on MCF-7 cells through downregulation of cyclin E2, p-ERK, and p21
Zhu et al. (2022) [28]	Investigation of the effect of fecal metabolites on the proliferative activity of tumor cells	HC11, ANA-1 and 4 T1	Flow cytometry, qRT-PCR, cytotoxicity assay, cell cocultures, enzyme-linked immunosorbent assay, cell migration, cell invasion, plaque cloning, and apoptosis analysis	0, 10, 20, 50, 100, 150, or 200 mM L-norvaline and 0.25 μM DOX for 24 h	The combination of L-norvaline and DOX ↓ the proliferation of BC cells

*GM* intestinal microbiota, *BC* breast cancer, *qRT-PCR* quantitative real-time polymerase chain reaction, *DOX* doxorubicin, *qPCR* quantitative polymerase chain reaction, *RT-PCR* reverse transcriptase polymerase chain reaction, *N/A* not applicable, *DMEM* Dulbecco’s modified Eagle’s medium, *IL-6* interleukin 6. ↑ means increase and ↓ means decrease

### Human studies

In human studies (Table 2), BC patients and controls were in all cases matched or similar in terms of age, body mass index (BMI), age at menarche and cancer grade and stage. Characterization of the microbiota in human trials was performed mainly from stool samples [26–40] although in some cases breast tissue was analyzed [41, 42]. Microorganisms were identified by sequencing regions of the 16S rRNA (ribosomal ribonucleic acid) gene [26–28, 30, 31, 33–40] or by shotgun metagenomic sequencing [32, 36]. Specifically, the 16S rRNA gene regions sequenced were V3–V4 [26–28, 30, 33, 34, 38], V4–V6 [42] or V4 [31, 35, 37, 39].

Studies characterizing GM mainly found that the microbial composition of BC patients differs from that of healthy controls [27, 28, 31–35, 37–39]. Therefore, GM dysbiosis should be associated with BC (Table 2). However, GM is not the only one that seems to play a role in this disease. Lately, the importance of the breast microbiota in BC is being investigated and, analogously to GM, studies performed on breast tissue samples found differences in the microbial composition in tumor tissue compared to adjacent healthy tissue [42] (Table 2). These findings are consistent with other studies [58–61] and a recent meta-analysis [62]. Moreover, GM can also be related to other breast lesions and its composition may differ from BC (Table 2). In this regard, the GM of patients with benign breast lesions and non-malignant breast disease was different from controls [38, 39]. In addition, the composition also varied between patients with BC and patients with benign breast lesions [38] or benign breast disease [40]. However, no differences were observed between patients with BC and non-malignant breast disease [39].

Measurement of α- and β-diversity showed controversial results (Table 2). Several studies found lower α-diversity in the GM of BC patients compared to controls [27, 31, 34, 38, 39], while others found increased α-diversity [28, 32] or no significant differences [33–35, 37]. α-Diversity was also lower in tumor tissue compared to adjacent healthy tissue [42], in patients with benign breast lesions and non-malignant breast disease compared to controls [38, 39] and in BC patients compared to patients with benign breast disease [40]. A decrease in GM diversity has been associated with a variety of pathological conditions, such as obesity, inflammatory bowel disease and autism [63, 64], and it has also been found in several types of cancer, such as colorectal cancer [65, 66]. Regarding β-diversity, changes in GM β-diversity allowed discrimination between BC patients and controls [32, 34, 35, 38, 39], patients with benign breast lesions, non-malignant breast disease and controls [38, 39], and even between BC patients and patients with benign breast lesions [38]. Nevertheless, Bilenduke et al*.* [37] and Bobin-Dubigeon et al*.* [27] found no significant differences in the β-diversity of the microbiota of patients when compared to controls. In the same line, Byrd et al*.* [39] did not find significant differences between BC patients and those with non-malignant breast disease and Esposito et al*.* [42] were unable to discriminate between tumor and adjacent healthy tissue based on microbiota β-diversity.

Contradictory data increase when comparing the results obtained in the studies that analyze the GM of specific patients (Table 2). In this regard, it should be noted that menopausal status is an important factor to take into account in BC since the disease is more aggressive and has a worse prognosis in premenopausal patients [67] and the risk of BC is increased in postmenopausal women because of the accumulation of endogenous estrogens. When investigating the GM of premenopausal women with BC, J. Zhu et al*.* [32] found no significant differences between the microbial species of patients compared to controls. However, Hou et al*.* [34] and He et al*.* [33] demonstrated the existence of dysbiosis in premenopausal patients although the GM imbalance was different in their population samples. Hou et al*.* [34] described an enrichment of *Anaerostipes* and *Bacteroides fragilis* and a reduction of *Bifidobacterium longum, B. bifidum* and *B. adolescentis* in the GM of patients. He et al*.* [33] observed an increased Firmicutes/Bacteroidetes ratio, as well as higher abundance of *Allisonella, Megasphaera, Pediococcus, Abiotrophia, Granulicatella, Clostridium_sensu_stricto, Serratia, Enhydrobacter, Fusobacterium* and *Slackia* genus, and lower abundance of *Clostridium_IV, Eubacterium, Terrisporobacter, Turicibacter, Intestinibacter, Butyricicoccus, Romboutsia, Providencia, Desulfovellario* and *Desulfovibrio*. Dysbiosis clearly characterized BC during postmenopause [31, 32, 34]. In spite of this, patients’ GM composition was highly variable. For instance, J. Zhu et al*.* [32] found significant differences in the relative abundance of 45 species, including increases in *Escherichia coli, Klebsiella sp_1_1_55, Prevotella amnii, Enterococcus gallinarum, Actinomyces* sp. *HPA0247, Shewanella putrefaciens, Erwinia amylovora* and *Acinetobacter radioresistens* and decreases in *Eubacterium eligens* and *Lactobacillus vaginalis*. Notably, *A. radioresistens* and E. *gallinarum* were weakly positively correlated with C-reactive protein expression and *S. putrefaciens* and *E. amylovora* correlated with estradiol levels, whereas *Actinomyces* sp. *HPA0247* was weakly negatively correlated with the number of T CD3+ and CD8+ cells. Hou et al*.* [34] observed an enrichment of *Proteobacteria* and *Klebsiella pneumoniae* and a decrease of *Akkermansia muciniphila* and *Phascolarctobacterium*. It is worth noting that Hou et al*.* [34] demonstrated the existence of differences in the GM of premenopausal and postmenopausal patients of BC. Based on this fact, they suggested that changes in the relative abundance of specific bacterial species could be used as potential universal BC biomarkers (high abundance of *Sutterella* and *Haemophilus parainfluenzae* and low abundance of *Faecalibacterium prausnitzii*, *Ruminococcus gnavus* and *Rothia mucilaginosa*), premenopause-specific BC biomarkers (increased abundance of *Anaerostipes* and *B. fragilis* and decreased abundance of *B. longum, B. bifidum* and *B. adolescentis*) or postmenopause-specific BC biomarkers (increased abundance of *Proteobacteria* and *K. pneumoniae* and decreased abundance of *A. muciniphila* and *Phascolarctobacterium*).

Among these potential universal BC biomarkers, *Sutterella* and *H. parainfluenzae* are pathogenic bacteria associated with autism, ulcerative colitis and oropharyngeal cancer [68–70]; *F. prausnitzii* is an important producer of SCFAs and its decrease serves as an indicator of dysbiosis [71], and decreased abundance of *R. gnavus* and *R. mucilaginosa* has also been found in other cancers [68, 72]. For premenopause-specific BC biomarkers, *Anaerostipes* has been linked to endometrial cancer, hepatocellular carcinoma and thyroid cancer [73–75]; *B. fragilis* contributes to the development of colorectal cancer [76]. Furthermore, *B. fragilis* is a resident bacterium in breast tissue and is able to colonize the gut to promote breast tumorigenesis and metastatic progression [16]. In addition, *Bifidobacterium* is a well-known probiotic. Finally, regarding postmenopause-specific BC biomarkers, *Proteobacteria* is a phylum enriched in pathogenic bacteria; *K. pneumoniae* is a pathogen that produces colibactin, a toxin that promotes the development of colorectal cancer [77]; the abundance of *A*. *muciniphila* is reduced in patients with metabolic diseases, such as obesity, diabetes and hypertension [78, 79] and *Phascolarctobacterium*, can produce SCFAs and is reduced in many types of cancer [73, 80].

The analysis of GM by He et al*.* [33] suggested that the enrichment in *Desulfovibrio* accompanied by a decrease in *Pediococcus* could have diagnostic value for premenopausal BC. *Desulfovibrio* genus has been associated with colon-related tumors [81] and it may contribute to inflammation and cardio-metabolic risk in BC [82], whereas *Pediococcus* exerts an anti-inflammatory role in the gut and has antiproliferative and anticancer activity on cervical and colon cancer cells [83, 84]. Moreover, *Pediococcus* is a genus that produces SCFAs. These metabolites seem to improve health status and their intestinal levels have also been reduced in premenopausal patients [33].

Interestingly, the GM of BC patients varied according to BC type, stage and grade, as well as based on other characteristics, such as BMI, percentage of total body fat, physical activity or age at menarche [29, 30, 39] (Table 2). For instance, Wu et al*.* [30] associated HER2-positive BC, overweight, obesity, increased total body fat, early menarche (less than or equal to 11 years) and a sedentary lifestyle with a decrease in α-diversity of GM and a different composition at phylum and genus level in BC patients. HER2-positive compared to HER2-negative patients were characterized by a decrease in *Firmicutes* (such as *Clostridium, Blautia, Coprococcus* and *Ruminococcus*) and an increase in *Bacteroidetes*. Early menarche compared to late menarche (greater than or equal to 12 years) was associated with a lower abundance of *Firmicutes* and a higher grade or stage of tumor correlated with enrichment of *Clostridium* and *Veillonella* and decreased abundance of *Erysipelotrichaecea.* Luu et al*.* [29] observed a similar pattern in overweight and obese patients. In this case, the GM of patients with high BMI was characterized by a decrease in *Firmicutes*, *F. prausnitzii, Blautia* sp*.* and *Eggerthella lenta*. Notably, *F. prausnitzii* exerts anti-inflammatory effects through the production of butyrate [85]. Thus, since both obesity and BC are associated with an inflammatory state, a significant decrease in this species could contribute to the development of the disease. The study also assessed the differences at the phylum, genus and species level depending on the stage and grade of BC. Patients in stage II/III had a higher total number of *Bacteroidetes, Clostridium coccoides* cluster, *C. leptum* cluster, *F. prausnitzii* and *Blautia* sp. compared to stage 0/I. Both *Clostridium coccoides* cluster and *C. leptum* cluster express β-glucuronidases which, as mentioned above, could contribute to the increase in systemic levels of free estrogens and favor the development of more severe clinical stages in patients with hormone-dependent BC. Finally, Luu et al*.* [29] found that an increased percentage of *Blautia* sp. correlated with a higher tumor grade, so this genus could be associated with poor prognosis. Based on these results, the GM profile could be used to define the molecular type, stage, and grade of BC. In addition, the relationship between this disease and GM dysbiosis suggests that GM alteration may be one of the mechanisms by which some risk factors contribute to the development of BC.

BC treatments, such as chemotherapy, affect the composition of the microbiome and, in turn, the microbiota may influence the effects of chemotherapy (Table 2). Terrisse et al*.* [36] described an association between GM β-diversity and tumor stage and grade, axillary node involvement and neurological side effects of chemotherapy. They also found different microbial composition and functional pathways depending on whether the patients had a favorable or unfavorable prognosis. Unfavorable prognosis was associated with *Streptococcus*, *Lachnospiraceae* (*Blautia wexlerae*), *Veillonella* (*V. parvula*), *Bacteroides* spp. (*B. uniformis*), *E. ramosum*, Enterobacteriaceae (*Klebsiella* spp.) and Clostridiaceae (*C. spiroforme, C. asparagiforme, C. boltae*); whereas a favorable prognosis was associated with Eubacteriaceae (*E. rectale*), *A. muciniphila*, *Defulfovibrio piger, Coprococcus* (*C. comes, C. catus*), *Collinsella, B. vulgatus* and Ruminococcaceae. The existence of a different microbial profile depending on the prognosis of the patients may indicate that the GM can be a predictive factor for the response to chemotherapy in BC patients. Furthermore, Terrisse et al*.* [36] demonstrated that the treatment was able to increase α-diversity and shift the microbial profile of patients with an unfavorable prognosis toward a microbial profile associated with a favorable prognosis. Thus, GM modulation could be used to promote an effective response to BC therapies. However, there is a need to determine which chemotherapeutic agent affect the microbiota and to validate its ability to inhibit unfavorable bacteria or enhance favorable commensals in future clinical trials. Similarly, Bilenduke et al*.* [37] found an association between cognitive impairment and depression symptoms with differences in the microbial composition of BC patients treated with chemotherapy. Specifically, they described a decrease in *Odoribacter* and an increase in *Clostridium*, *Eggerthella* and Erysipelotrichi. In this regard, certain drugs used in chemotherapy can contribute to a state of systemic inflammation, but these effects can also be attributed to various microorganisms. Therefore, taking into account that chemotherapy is capable of altering GM, it could potentiate the inflammatory effects of therapies too. For instance, *Odoribacter* is a butyrate-producing genus (anti-inflammatory SCFA), while Erysipelotrichi is associated with intestinal inflammation and activation of inflammatory pathways [86, 87]. Accordingly, Bilenduke et al*.* [37] found a large decrease in the abundance of the genus *Akkermancia* in BC patients. Low abundance of this genus is related to pathologies such as irritable bowel syndrome and is associated with loss of intestinal barrier integrity and inflammation [88].

The results obtained when characterizing the composition of the GM largely differ among the different studies included (Table 2), as previously described for specific groups of patients. Q. Zhu et al*.* [28] assessed GM alterations in patients compared to controls and observed in patients an increase in the relative abundance of *Rothia, Actinomyces, Lautropia*, *Centipeda, Corynebacterium, Anaeroglobus*, *Selenomonas, Fretibacterium* and *Tannerella*, whereas a decrease *Subdoligranulum*. The abundance of the *Actinomyces* and *Rothia* genus has been linked to the development of some cancers, such as colorectal cancer for *Actinomyces* [89] and squamous cell lung carcinoma for *Rothia* [90]. Moreover, both *Actinomyces* and *Rothia* genus have been negatively associated with levels of L-norvaline, a metabolite that combined with DOX could counteract carcinogenesis [91, 92]. Bobin-Dubigeon et al*.* [27] found that the GM of patients with early-stage BC was characterized by a relative enrichment in *Firmicutes*, *Clostridium* cluster IV and cluster XIVa, *Blautia*, *Clostridium* XVIII and *Lachnospira*; as well as a decreasse in *Bacteroidetes*, *Bifidobacterium* sp., *Odoribacter* sp., *Butyricimonas* sp. and *Coprococcus* sp. *Clostridium* cluster IV and *Clostridium* cluster XIVa express β-glucuronidases which, as previously mentioned, are enzymes that contribute to increased serum levels of free estrogens and, consequently, BC risk. Similarly, *Coprococcus* sp., *Butyricimonas* sp. and *Odoribacter* sp. are SCFA-producing genera, and their decrease has also been observed in colorectal cancer [93], non-Hodgkin lymphoma [94] and colorectal cancer [95], respectively. Bobin-Dubigeon et al*.* [27] also found a decrease in *Bifidobacterium* sp. (a genus that typically includes probiotic microorganisms related to the maintenance of human health status) in patients. The analysis of obese and overweight patients by Smith et al*.* [35] revealed higher levels of the genus *Allobaculum* and lower levels of *Lysobacter* in the GM of patients, while *Agrobacterium* was the predominant genus in the GM of controls. Saud Hussein et al*.* [41] compared the composition of the breast microbiota and observed growth of *E. coli* and *Staphylococcus aureus* in the breast tissue of BC patients but were unable to detect growth in samples from women with benign breast lesions. Nevertheless, Esposito et al*.* [42] detected an increased abundance of *Proteobacteria* and *Firmicutes* and a decrease in *Actinobacteria* (especially *Propionibacterium acnes*) in tumor breast tissue. These results are in agreement with the study by Thompson et al*.* [96]. *Propionibacterium acnes* is an opportunistic pathogen whose role in human health has not yet been established. Some authors support its antitumor effect in BC [97], while others suggest that it is involved in implant-associated infections [98]. Unlike the tumor tissue, *Propionibacterium* and *Pseudomonas* predominated in healthy adjacent tissue [42].

Finally, regarding other breast lesions (Table 2), Z. Ma et al*.* [38] investigated the composition of the GM in patients with BC and benign breast lesions. The authors established the increase of *Porphyromonas, Prevotella, Peptoniphilus* and *Megamonas* as an indicator of BC and the increase of *Escherichia, Lactobacillus* and *Coprobacillus* levels were associated with benign breast lesions. *Porphyromonas* and *Prevotella* have also been identified as potential biomarkers of postmenopausal BC [32, 99] and have been associated with colorectal cancer and precancerous adenomas [100, 101]. Similarly, *Peptoniphilus* genus was enriched in HER2-positive and triple-negative subtypes of BC [102]. Additionally, within the genus *Escherichia*, *E. coli* is capable of producing DNA mutagens, such as colibactin genotoxin, and inducing tumorigenesis [103]. Although Byrd et al*.* [39] found no significant differences between the microbiota of patients with BC and non-malignant breast disease, BC was positively associated with *Bacteroides* and *Ruminococcaceae* and negatively associated with Romboutsia*, Coprococcus* and *Faecalibacterium*. J. Ma et al*.* [40] identified 59 members of the microbiota that differed in abundance between patients with BC and benign breast disease. BC patients displayed a lower relative abundance of *Firmicutes* (*Faecalibacterium*) and *Bacteroidetes* and increased abundance of *Verrucomicrobia*, *Proteobacteria*, *Actinobacteria*, *Bacillus*, *Enterobacter* and *Staphylococcus.* Considering that many breast lesions can give rise to precursors of BC or be risk markers for the disease, the identification of microbial profiles associated with these lesions could contribute to early detection and help prevent BC development.

The entire gut microbiome was altered in BC patients (Table 2). Several studies included in this review have demonstrated that diverse metabolic pathways and particular metabolites can distinguish the microbiota of different patient groups. Zhu et al*.* [28] found a relationship between differences in the composition of the GM and its metabolites in patients and controls. For example, enrichment of *Rothia* and *Actinomyces* in patients was positively associated with the presence of 4-methylcatechol and guaiacol, but negatively associated with norvaline. Byrd et al*.* [39] observed an association between BC and non-malignant breast disease with taxa involved in estrogen metabolism and immune homeostasis. Similarly, J. Ma et al*.* [40] identified 26 metabolic pathways that differed between the microbiota of BC patients or benign breast disease. Additionally, the abundance of some genes and the activation of metabolic pathways were also related to the GM composition of premenopausal and postmenopausal BC patients. In this regard, Zhu et al*.* [32] identified an increase in genes related to PTS, secretion, vitamin B12 transport and manganese/iron systems in premenopausal and postmenopausal patients compared to controls. In addition, postmenopausal patients showed higher expression of several genes involved in lipopolysaccharide biosynthesis and lower expression of genes related to butyrate synthesis. Nevertheless, Hou et al*.* [34] revealed that premenopausal patients had a microbiota enriched in bacteria involved in steroid-related and oncogenic pathways, whereas the microbiota of postmenopausal patients was mainly composed of bacteria involved in chemical carcinogenesis and aldosterone-related pathways. He et al*.* [33] assessed premenopausal patients and observed a lower proportion of SCFA-producing bacteria and a decrease in the levels of these metabolites, especially butyrate. The microbiota of postmenopausal patients was characterized by an enrichment of metabolic pathways of immune diseases based on the study by Goedert et al*.* [31].

Taken together, BC is associated with an imbalance in the gut and breast microbiome. This dysbiosis affects both the diversity and abundance of specific microorganisms and their metabolites and varies according to clinical features (such as stage, grade, and molecular subtype of BC) and host factors (such as menopausal status, age at menarche, overweight, obesity and physical activity). In addition, the interaction between microorganisms and the development and progression of cancer is very complex, and a variety of bacteria or their metabolites can promote or inhibit the development of BC. In addition to this fact, the heterogeneity of the results reported does not allow a specific microbial profile to be associated with BC. In general, it seems that BC is related to a lower microbial diversity, an enrichment of genera with harmful effects (associated with various types of cancer, pathologies and inflammation or producers of β-glucuronidases and toxins), such as *Clostridium* and *Bacteroides,* and decreased in genera beneficial to health, such as *Faecalibacterium, Bifidobacterium* and *Akkermansia*. Furthermore, this disease can be related to a significant decrease in SCFA-producing bacteria and lower levels of these metabolites.

### Animal model studies

Modulation of GM could be a useful strategy to enhance the efficacy of therapies against BC. This review includes some studies in animal models that support this hypothesis (Table 3). Shi et al*.* [45] highlights the implications of combined therapy of transforming growth factor-β (TGF-β) inhibitors, such as galunisertib, with *Escherichia coli* Nissle 1917 in the prevention and treatment of BC. Oral administration of *E. coli* Nissle 1917, a probiotic with beneficial effects on intestinal immune homeostasis, enhanced the effect of galunisertib by modulating the GM and the tumor immune microenvironment. Specifically, *E. coli* Nissle 1917 caused an increase in the abundance of *Alistipes shahii*, *A. muciniphila*, *Bacteroides thetaiotaomicron*, *B. acidifaciens* and *Lactobacillus johnsonii*; and a decrease in the abundance of *Clostridium* spp.

Di Modica et al*.* [26] determined that certain commensal bacteria may contribute to the efficacy of trastuzumab through modification of the tumor microenvironment. In their model of HER2-positive BC, antibiotic administration reduced the efficacy of trastuzumab by altering the GM by reducing the abundance of *Clostridiales* (*Lachnospiraceae*), *Actinobacteria* (*Coriobacteriaceae*), *Turicibacteraceae* and *Bacteroidetes* (*Prevotellaceae*). In addition, the GM composition of HER2-positive patients had an impact on the activity of trastuzumab, as the transfer of fecal microbiota from patients with different treatment responses to mice recapitulated the results observed in patients. Furthermore, Terrisse et al*.* [36] discovered that GM composition influences the progression of BC and the anticancer effects of cyclophosphamide (CTX) since mice displayed different microbial compositions depending on tumor development and CTX activity. The GM of mice with slow tumor progression was enriched on species present in the feces of patients in stage I or no axillary node involvement pre- or post-chemotherapy (*Eubacterium rectale, E. eligens*, *A. muciniphila*, *B. longum, C. aerofaciens* and *Alispites shahii*). On the contrary, mice that received fecal microbiota transplantation from patients associated with an unfavorable prognosis (*B. uniformis, B. xylanivolvens, B. intestinalis*) showed rapid disease progression. In addition, the tumor-killing activity of CTX was lower in mice with fecal microbiota transplant from patients compared to mice with fecal microbiota transplant from controls. These studies suggest that some microbial populations could serve as biomarkers to predict treatment response.

Dietary intervention may represent a promising strategy for the prevention and treatment of BC, as the intake of a wide range of compounds is able to regulate GM homeostasis and influence tumor progression in animal models (Table 3). Paul et al*.* [43] found that genistein, an isoflavone derived from soy products with anticancer properties, could increase tumor latency and reduce tumor growth in ER-negative mice by modulating GM. In particular, the GM of genistein-treated mice was characterized by an increase in *Lactococcus* and *Eubacterium* genus, members of Lachnospiraceae and Ruminococcaceae family and *Verrucomicrobia* phylum (such as *A. muniphila*). Daucosterol linolenate (DLA), daucosterol linoleate (DL) and daucosterol palmitate (DP), three phytosterols present in sweet potato, inhibited tumor growth in MCF-7 cell xenograft nude mice by altering the expression of tumor markers and cancer-related proteins, modulating GM and production of SCFAs. In particular, the three phytosterols reversed tumor-induced dysbiosis by increasing *Bacteroidetes* richness and decreasing *Firmicutes* richness, modulating GM diversity at family and genus level, and promoting butyric or acetic and butyric acid production by DL and DP, respectively [48]. Fucoidan, a complex sulphated polysaccharide obtained from brown algae, favored the development of a more diverse gut microbiome through an increase in the *Bacteroidetes*/*Firmicutes* ratio and an increase in SCFAs producers such as *Prevotella* in a rat model of BC. Furthermore, fucoidan repaired intestinal barrier function by promoting the expression of tight junction proteins (Zonula occludens-1, claudin-1 and claudin-8) and the levels of phosphorylated p38 MAPK and ERK1/2 [44]. The administration of *Poria cocos* fungus extracts exerted a similar effect in triple-negative BC mice. In this case, the change in the structure of the GM resulted in an increase in beneficial bacteria, such as *Lactobacillus*, *Bifidobacterium* and *Blautia,* and a decrease in sulfate-reducing bacteria, such as *Desulfovibrio* and bacteria, associated with inflammation, such as *Mucispirillum*, *S24-7* and *Staphylococcus* [47]. Finally, Li et al*.* [49] determined that maternal n-3 polyunsaturated fatty acids reduced the risk of BC in the offspring by modulating the GM and reducing levels of the proinflammatory factors interleukin 1β (IL-1β), IL-6 and tumor necrosis factor α (TNF-α). In particular, n-3 polyunsaturated fatty acids caused an increase in α-diversity and relative abundance of *Akkermansia, Lactobacillus* and *Mucispirillum*. *Mucispirillum* was also positively associated with IL-10 levels, whereas *Akkermansia* was negatively associated with IL-6.

Although bacteria are the most studied members of the human microbiota, they are not the only microorganisms that can be involved in BC and its treatment. Shiao et al*.* [50] discovered that the mycobiome plays an opposite role to that of the bacteriome in the efficacy of radiotherapy against BC, by modulating the antitumor immune response (Table 3). While the decrease in gut fungi favors the efficacy of radiotherapy, the decrease in bacteria reduces the response to radiotherapy and is related to the overgrowth of commensal fungi.

Several studies in mouse models have addressed the link between alterations in GM in obesity associated with BC, the existence of previous dysbiosis and its relation with cancer prognosis and the effects of the tumor on the microbiota and intestinal barrier function (Table 3). Hossain et al. [51] used an immunocompetent mouse model of triple-negative BC to conclude that there is an association between Western diet-induced obesity and increased tumor growth, which is consistent with previous studies, suggesting that obesity favors the risk of this subtype of BC [104]. Similarly, obesity was associated with a loss of microbial diversity, a decrease in the *Bacteroides*/*Firmicutes* ratio (particularly *Alistipes*) and an alteration in bacterial metabolic pathways. This decrease in microbial diversity is also consistent with compelling data available in the existing literature [105]. Finally, Hossain et al*.* [51] demonstrated that the similarity of bacterial communities based on taxonomic profiles and the variability of functional profiles depended on obesity, although the latter was also explained to a lesser extent by the presence of the tumor and the obesity–tumor interaction.

Loman et al*.* [52] discovered that orthotopic mammary tumors compromise the intestinal barrier function by altering the microbiome, highlighting a lower abundance of *Lactobacillus* and increased *Bacteroides*. Consequently, they increase the translocation of enteric bacteria and cause systemic inflammation including splenomegaly, increased splenic bacterial load and proinflammatory cerebral and splenic cytokines. This may explain the gastrointestinal (diarrhea and nausea) and cognitive (anxiety and depression) symptoms associated with the tumor. In this context, *Lactobacillus* supplementation could help to alleviate these symptoms, as this probiotic genus is known to improve intestinal barrier function, regulate the immune system and influence intestinal motility [106, 107]. Furthermore, Rosean et al*.* [46] identified pre-existing dysbiosis of the commensal microbiota as a host-intrinsic regulator of tissue inflammation, myeloid recruitment, fibrosis, and tumor cell dissemination in ER/PR-positive BC. Therefore, these authors demonstrated that commensal dysbiosis contributes to the spread and aggressiveness of BC and that dysbiosis could be a biomarker or therapeutic target to reduce inflammation within the tissue microenvironment.

GM characterization in animal models was performed on stool samples and microbial composition was determined mainly by sequencing regions of the 16S rRNA gene [26, 43–52] or by shotgun metagenomic sequencing [36]. Sequenced 16S rRNA gene regions were V3-V4 [26, 44, 45, 47–49] or V4 [43, 51, 52]. In addition, in the case of the mycobiome, the composition of gut fungi was determined by sequencing the ITS1 gene [50].

### In vitro studies

In vitro studies with BC cell lines (Table 4) highlight the potential therapeutic use of specific members of the microbiota or their metabolites in BC treatment. Ma et al*.* [40] discovered that *Faecalibacterium prausnitzii* prevents MCF-7 cells growth by inhibiting the IL-6/STAT3 pathway. This signaling pathway is hyperactivated in many cancers and its hyper-activation is associated with poor prognosis. In addition, in the tumor microenvironment, the IL-6/STAT3 pathway promotes tumor cell proliferation, survival, invasiveness and metastasis, while suppressing the antitumor immune response [108]. Consequently, *F. prausnitzii* could act as a probiotic in BC by inhibiting tumor growth and stimulating antitumor immunity. In the same cell line, An et al*.* [54] determined that extracellular vesicles derived from *Klebsiella pneumoniae* potentiate the antihormonal effect of tamoxifen by downregulating cyclin E2, p-ERK and p21. Furthermore, various metabolites produced by the GM also showed inhibitory effects on BC cell lines. Zhu et al*.* [28] found that L-norvaline can inhibit BC cell proliferation when combined with DOX. Moreover, He et al*.* [33] demonstrated that sodium propionate and sodium butyrate inhibit the activity of SKBR3 and MCF-7 cells in a dose-dependent manner.

Finally, some studies in BC cell models suggest that disruption of lipid rafts may be a key factor in BC cell proliferation and apoptosis. Bobin-Dubigeon et al*.* [53] found that GM interacts with the lipid metabolism of enteric cells and influences the behavior of MCF-7 cells. Since lipid metabolites can reach mammary cells through systemic circulation, they could affect BC. The authors used a basolateral medium of Caco-2 cells preincubated with patients or control fecal fluid and concluded that preincubation with patient or control fluid differentially affected MCF-7 cells viability. Fecal water valerate, a SCFA, was independently associated with a decreased ability of the Caco-2 cell medium to induce MCF-7 cell proliferation. In addition, MCF-7 cells viability was positively related to the percentage of *Bifidobacterium* sp. in the fecal water incubated with Caco-2 cells. Finally, regarding the expression of genes related to lipid metabolism, they found a positive relationship between the expression of the *Apo AIV* gene and acetate, butyrate, propionate, and the percentage of Bacteroidetes. Moreover, the percentage of *Blautia* sp. was negatively correlated with the expression of the *LXR* (*liver X receptor*) gen. *Apo AIV* and *LXR* genes regulate the synthesis of apolipoproteins, proteins that are involved in the release of cholesterol from BC cells, which is associated with reduced cell viability. Considering the above, a relationship has been found between the abundance of *Blautia* sp*.* and the severity of BC, this genus may influence tumor development through its negative effect on the *LXR* gene [53].

## Limitations of the study

This literature review has some limitations. The bibliographic search was performed in a single database, which limited the incorporation of publications. Several of the included trials were carried out with a small number of samples and assessed the GM in patients with different characteristics, which limits the robustness of the conclusions. Additionally, the reported results show great heterogeneity, possibly due to biological factors (such as age, individual genetic variation, ethnic origin, geographic location, and dietary habits) that influence the composition of the microbiota, and the variability of analysis methods (hypervariable regions of the 16S rRNA gene, type and sequencing platform, bioinformatics tools, etc.). Finally, most of the articles identify the members of the GM at the genus level or higher levels, which precludes the establishment of a link of specific species with BC.

Finally, we are aware of recent changes in bacterial taxonomy and nomenclature. However, we have kept the nomenclature used by the authors for better traceability of the works mentioned in this review.

## Conclusions

Based on the literature included in this work, some clear conclusions can be drawn. GM differs between BC patients and healthy women. Therefore, BC may be associated with microbiota dysbiosis. This imbalance affects microbial diversity and the abundance of particular microorganisms and their metabolites. Similarly, it varies depending on the molecular type, stage and grade of cancer, as well as based on the state of menopause, age at menarche, BMI and physical activity of the patients.

BC seems to be characterized by a loss of microbial diversity, an enrichment of genera with deleterious effects (related to cancer, pathologies and inflammation or producers of β-glucuronidases and toxins), such as *Clostridium* and *Bacteroides*, and a decrease in genera beneficial for the health as *Faecalibacterium*, *Bifidobacterium* and *Akkermansia.* In addition, BC can be related to a decrease in SCFA-producing bacteria and the levels of these metabolites. However, it has not been possible to identify a specific microbial profile as a non-invasive biomarker.

Studies in humans, animal models and in vitro indicate that GM may influence the efficacy of BC therapies through modulation of the tumor immune microenvironment, probably also being partly responsible for the treatment side effects.

Studies performed on animal models and in vitro cell cultures demonstrate the potential use of various microorganisms (such as *Escherichia coli* Nissle 1917 and *Faecalibacterium prausnitzii*) as probiotics to promote the efficacy of BC therapies or directly affect tumor development. Moreover, a variety of microbial metabolites (such as SCFA, such as butyrate, propionate and valerate) have been proposed as prebiotics.

Large-scale studies, especially clinical trials with standardized protocols, and the application of proper mathematical approaches to calculate adequately the microbiome abundances are necessary to confirm the association between microbiota and BC and to discover the potential clinical applications of the microbiota in the prevention and early diagnosis of BC, as well as therapeutic interventions [109].

The existence of biomarkers based on the composition of the human microbiota would provide a new method for the diagnosis of non-invasive BC. Furthermore, the ability of the microbiota to modulate therapies would allow the development of more effective therapeutic strategies, ultimately contributing to reducing mortality and prevalence of BC. Nevertheless, further research is needed to shed light on the relevance of microbiota modulation for BC treatment.

## Data Availability

Not applicable.

## Abbreviations

DNA: Deoxyribonucleic acid
SCFA: Short-chain fatty acids
rRNA: Ribosomal ribonucleic acid
BC: Breast cancer
CTLA-4: Cytotoxic T cell antigen 4
CTX: Cyclophosphamide
DLA: Daucosterol linolenate
DL: Daucosterol linoleate
DP: Daucosterol palmitate
DOX: Doxorubicin hydrochloride
HER2: Human epidermal growth factor receptor 2
ICI: Immune checkpoint inhibitors
IL: Interleukin
BMI: Body mass index
GM: Gut microbiota
NF-κB: Nuclear factor kappa B
NLR: NOD-like receptors
PAMP: Pathogen-associated molecular pattern
PD-1: Programmed cell death receptor 1
PD-L1: Programmed cell death ligand 1
PD-L2: Programmed cell death ligand 2
PRR: Pattern recognition receptors
qPCR: Quantitative polymerase chain reaction
ER: Estrogen receptor
PR: Progesterone receptor
RT-qPCR: Real-time quantitative polymerase chain reaction
sIgA: Immunoglobulin A secretory
TGF-β: Transforming growth factor beta
TLR: Toll-like receptor
TNF-α: Tumor necrosis factor alpha
